# Preoperative prediction of extramural venous invasion in rectal cancer by dynamic contrast-enhanced and diffusion weighted MRI: a preliminary study

**DOI:** 10.1186/s12880-022-00810-9

**Published:** 2022-04-28

**Authors:** Weiqun Ao, Xian Zhang, Xiuzhen Yao, Xiandi Zhu, Shuitang Deng, Jianju Feng

**Affiliations:** 1 Department of Radiology, Tongde Hospital of Zhejiang Province, Hangzhou, Zhejiang Province China; 2 Departments of Radiology, Zhuji Affiliated Hospital of Shaoxing University, Zhuji People’s Hospital, No. 9 Jianmin Road, Zhuji, 311800 Zhejiang Province China; 3 Department of Ultrasound, Shanghai Putuo District People’s Hospital, Shanghai, China

**Keywords:** Rectal cancer, MRI-predicted, Extramural venous invasion, Diffusion-weighted imaging, Prognosis

## Abstract

**Background:**

To explore the value of the quantitative dynamic contrast-enhanced magnetic resonance imaging (DCE-MRI) and diffusion-weighted imaging (DWI) parameters in assessing preoperative extramural venous invasion (EMVI) in rectal cancer.

**Methods:**

Eighty-two rectal adenocarcinoma patients who had underwent MRI preoperatively were enrolled in this study. The differences in quantitative DCE-MRI and DWI parameters including Krans, Kep and ADC values were analyzed between MR-detected EMVI (mrEMVI)-positive and -negative groups. Multivariate logistic regression analysis was performed to build the combined prediction model for pathologic EMVI (pEMVI) with statistically significant quantitative parameters. The performance of the model for predicting pEMVI was evaluated using receiver operating characteristic (ROC) curve.

**Results:**

Of the 82 patients, 24 were mrEMVI-positive and 58 were -negative. In the mrEMVI positive group, the Ktrans and Kep values were significantly higher than those in the mrEMVI negative group (*P* < 0.01), but the ADC values were significantly lower (*P* < 0.01). A negative correlation was observed between the Ktrans vs ADC values and Kep vs ADC values in patients with rectal cancer. Among the four quantitative parameters, Ktrans and ADC value were independently associated with mrEMVI by multivariate logistic regression analysis. ROC analysis showed that combined prediction model based on quantitative DCE parameters and ADC values had a good prediction efficiency for pEMVI in rectal cancer.

**Conclusion:**

The quantitative DCE-MRI parameters, Krans, Kep and ADC values play important role in predicting EMVI of rectal cancer, with Ktrans and ADC value being independent predictors of EMVI in rectal cancer.

## Background

As the most common gastrointestinal malignancy, rectal cancer seriously threats to human health. The morbidity of the rectal cancer has gradually increased over the last decade all over the world [1, 2].

In rectal cancer, extramural venous invasion (EMVI) is defined as “the presence of malignant cells within blood vessels beyond the muscularis propria”, which is present in approximately one-third of patients with rectal cancer [3]. EMVI is not only one of the main risk factors of recurrence and synchronous/metachronous distant metastases, but also an independent indicator of a poor prognosis [4, 5]. EMVI has been included as an important imaging parameter prior to neoadjuvant therapy in the National Comprehensive Cancer Network guidelines for rectal cancer [6]. Therefore, detection of EMVI is critical for accurate preoperative risk stratification and influences treatment decision making.

However, EMVI is based on pathological evaluation of the intraoperative specimen, i.e. this pathological finding can be obtained only after surgery. If such information could be acquired before surgery, or even during preoperative diagnostic evaluation, there is potential for improved decision making of an optimal treatment plan and for better prediction of outcomes [7]. On the other hand, relying entirely on histological specimen, may underestimate the detection sensitivity of EMVI [8]. Instead, magnetic resonance imaging (MRI) allows us to observe the entire rectum and perirectal condition, so it is one of the best non-invasive methods which provides great advantages in detecting EMVI of rectal cancer [8, 9]. The recognition of EMVI based on functional MRI (fMRI) might be comparable to histopathological identification in rectal cancer [10, 11].

MR-detected EMVI, denoted as mrEMVI, was reported for the first time by Smith et al. in 2008 [10]. It is defined as “serpiginous extension of tumor signal within a vascular structure, leading to expansion of the vein by tumor signal and irregular contouring of the vessel border”. The status of mrEMVI was scored from 0 to 4, with stages 3 and 4 recorded as positive and stages 0, 1 and 2 as negative (Table 1). Some research has shown that mrEMVI was a strong predictive factor of poor prognosis [11, 12]. Recently, fMRI has been shown to provide good feasibility and diagnostic accuracy for the pre-surgical evaluation of mrEMVI [3, 13].

**Table 1:** 5-point scale classification on MRI for detection of EMVI

mrEMVI score	MRI features
0	No vessels adjacent to areas of tumor penetration
1	Minimal extramural tumor stranding/nodular extension, but not adjacent vascular structure
2	Extramural stranding in the vicinity of extramural vessels, but that are normal in caliber; no exact tumor signal within vessels
3	Intermediate tumor signal intensity within a mesorectal vascular structure that have contour and caliber only slightly expanded
4	Obvious irregular vessel contour or nodular expansion of vessels by serpiginous extension of the definite tumor signal intensity in large anatomic vessels

Diffusion-weighted imaging (DWI) and Dynamic contrast-enhanced magnetic resonance imaging (DCE-MRI) are widely used scanning techniques in clinical research and practice. As a functional imaging technology, DCE-MRI can reflect the attributes of tumor microvasculature that changes in hemodynamics and integrates morphology by quantifying parameters related to permeability, perfusion, and tumor micro-angiogenesis [14]. Quantitative DCE-MRI parameters and apparent diffusion coefficient (ADC) values were reported in close correlation with clinical, histological grade, response to neoadjuvant chemoradiotherapy (CRT) and prognostic factors of various tumors [15–17]. DWI implys the diffusion movement of water molecules by counting the ADC value of the tumor [18, 19]. Some studies [20–22] have shown that the ADC value of rectal cancer was closely related to tumor stage, malignancy and prognosis. Herein, the ADC value should be considered a sensitive image biomarker of rectal cancer.

To the best of our knowledge, the existing literature [23–25] mainly focused on preoperative therapy response, staging, and prognostic assessment in rectal cancer by DCE-MRI or DWI. Few studies have been carried out focusing on the prediction and prognostic impact evaluation of EMVI by quantitative DCE-MRI and DWI in rectal cancer.

This study aimed to explore the diagnostic performance of quantitative DCE-MRI (Ktrans, Kep and Ve) and DWI (ADC value) for the assessment of EMVI in patients with rectal cancer, so as to provide the basis for selecting optimal treatment management strategies and predicting prognosis.

## Methods

### Patients

**Fig. 1:**
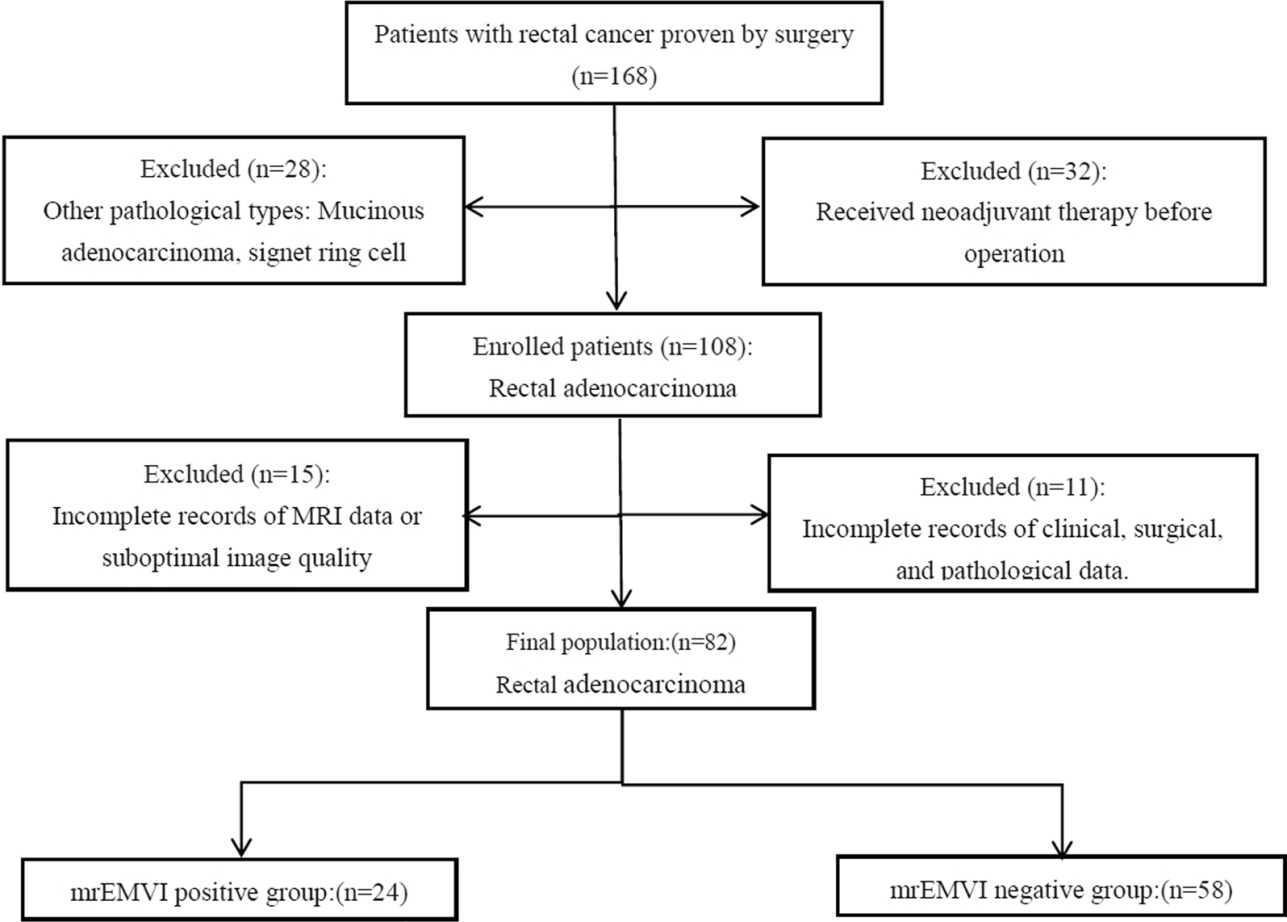
Flow diagram of enrolled patients

This retrospective study was approved by the Ethics of Committees of Tongde Hospital of Zhejiang Province and informed consent for this retrospective study was waived. All of the procedures were performed in accordance with the Declaration of Helsinki in 1964 and relevant policies in China. From March 2016 to December 2020, a total of 82 patients with rectal adenocarcinoma confirmed by surgery and pathology in our hospital were enrolled. All patients underwent MRI and DCE-MRI scanning before surgery. Our inclusion criteria were: histopathologically confirmed rectal adenocarcinoma, without pelvic surgical history (pelvic surgical clips may cause MRI scanning artifacts). Exclusion criteria were: incomplete MRI examinations and incomplete records of imaging, scan sequences, clinical, surgical, and pathological data; Patients underwent neoadjuvant therapy before surgery; Other pathological types: Mucinous adenocarcinoma, melanoma and signet ring cell carcinoma (Fig. 1).

### Histopathologic evaluation

Histopathological information, including histological grade, T stage, lymph node (N) stage, infiltration depth of tumor, tumor circum-involvement ratio (CIR), location (high, middle and low rectum), and Ki67 index expression, was obtained from pathological reports and confirmed by two pathologists with more than 15 years of experience in pathology. Discrepancies between the readers were resolved by consensus after joint re-evaluation of the images or specimens. T stage was categorized as T1, T2, T3 and T4, respectively. Lymph node stage was categorized as positive (including N1, metastasis in 1–3 nodes; and N2, metastasis in four or more nodes) or negative (N0: no metastasis in regional nodes). Histological outcome was categorized into three grades (well, moderately, and poorly differentiated). Positive Ki67 labeled cells were counted through light microscopy, with a distinct brown staining in the cytoplasm of neoplastic cells. Those positive ratios and positive cells were calculated. The regions with the largest number of positive tumor nuclei were selected for analysis. Criteria for pEMVI positive: HE staining revealed the presence of a rounded mass of tumor tissue within an endothelium-lined space beyond the muscularis propriain and tumor cells invade the lumen of blood vessels or lymphatic vessels slides. pEMVI negative: HE staining revealed tumor cells do not surround or invade the lumen of blood vessels or lymphatic vessels. A pathologic report of EMVI (positive, negative) was obtained for each patient.

### MRI protocol

After fasting for eight to twelve hours, all patients underwent MRI plain examinations and MRI enhancement scanning on 3.0 Tesla Siemens scanner (Siemens Magnetom Verio; Siemens Medical Systems, Erlangen, Germany), using phased-array body coil before operation. To reduce rectal spasm, they received injection of 20 mg hydrochloride hyoscine butylbromide (Minsheng Pharmaceutical, China). Scanning sequence showed in Table2.

**Table 2:** MRI scanning parameters in this study

Parameters	HR-T2WI	DWI	CE-T1
TR,ms	3200	9700	5.1
TE,ms	81	93	1.7
FOV,mm	200 × 200	250 × 250	260 × 250
Thickness,mm	3	3	3
Matrixs	256 × 256	250 × 250	138 × 192
b values	-	0, 800, 1500	-

*CE-T1* contrast-enhanced T1WI, *HR-T2WI* high resolution T2WI

DCE-MRI scanning was performed with breath-hold multiphase liver acceleration volume acquisition sequence. A bolus of Gadopentetate meglumine (Beilu Pharmaceutical, China) at a constant dose of 0.1 mmol/kg was power injected into the dorsal metacarpal veins, followed by saline flush at a rate of 2.5 mL/s for all patients (20 mL). The DCE-MRI obtained 30 axial slices during each phase, a total of 35 consecutive automatic scans were obtained. The whole scanning time lasted about 35–40 min.

### Image analysis

MRI images interpretation was retrospectively reviewed by two radiologists with more than 15 years of experience in abdominal imaging. The radiologists were blinded to the clinical data and pathological results of all the patients. Discrepancies between the readers were resolved by consensus after joint re-evaluation of the images. The examinations were reviewed in random order, with a time interval of at least 1 month.

According to the 5-point scale classification on MRI for detection of EMVI specified by Smith et al. [10], a score of 0–2 point was marked as mrEMVI-negative, and 3–4 point marked as -positive. Orderly viewing of the MRI sequence: T2WI revealed the serpiginous extension of signal intensity of the tumor within a mesorectal vascular structure, leading to vessel expansion and irregular contouring of the vessel border; DWI showed high signal in vasculature; intraluminal filling defect was visible. Contrast-enhanced T1WI (CE-T1) showed that vascular cavity was replaced by filling defect or tumor signal shadow.

Quantitative parameter measurement: The ROI was manually traced on maximum dimension of the visible tumor, while avoiding blood vessels, calcification, necrosis and cystic portions, on ADC map and DCE-MRI, respectively. The T2 weighted and DW images were used as reference to ensure accurate positioning of ROI. Quantitative DCE-MRI and ADC value were measured by a radiologist with 15 years of experience in abdominal imaging (Doctor A) and confirmed by another doctor radiologist (Doctor B) with 20 years of experience in abdominal imaging. Discrepancies between the readers were resolved by consensus after joint re-evaluation of the images. The ADC value was calculated by using the Functool software on a Siemens workstation (syngo MultiModality Workplace, SIEMENS, Germany). Quantitative DCE-MRI parameters were acquired using a tissue 4D software (MMWP version workstation, SIEMENS, Germany). The ADC value, volume transfer constant (Ktrans), extracellular extravascular volume fraction (Ve) and reverse reflux rate constant (Kep) parameters were measured and recorded.

DCE-MRI post processing: Quantitative DCE-MRI data analysis was performed using Tissue 4D software (Syngo MultiModality Workplace, Siemens Healthcare, Germany). First, DCE images were loaded to the workplace and motion correction was made. Second, ROIs were drawn on tumors, and then a time-signal curve was built. Third, the volume of interest (VOI) was manually traced along the edges of the tumors while avoiding bladder on DCE-MRI images. To calculate the quantitative parameters of the VOI, an input function with the smallest value of chi-square was selected. Fourth, the ROIs were copied to parametic map, and the mean quantitative perfusion parameters (Ktrans, Kep and Ve) were automatically calculated.

To ensure the reliability of measurements, these cases were also assessed by another radiologist (X.Z1). Interobserver agreements for quantitative parameters of DCE-MRI were assessed by intra-class correlation coefficients (ICC) with 2-way random method between the two radiologists (W.A and X.Z1).

### Statistical analysis

Statistical analyses were performed using SPSS software (IBM Corporation, version 22.0). Continuous variables with normal distribution were presented as mean ± standard deviation, and categorical variables were presented as frequencies and percentages. Kappa test was used to assess consistency between MRI and pathology analysis of EMVI in rectal cancer. Poor agreement (Kappa < 0.40), Moderate agreement (0.40 ≤ Kappa < 0.70), and good agreement (Kappa ≥ 0.70) were defined. If the consistency was good, the averages of the measurements were used for further statistical analysis; If the consistency was poor, the measurement data of a third radiologist were added. Interobserver agreements for ADC values and DCE-MRI parameters were analyzed by ICC. The ICC was defined as follows: poor (< 0.20), fair (0.20–0.40), moderate (0.41–0.60), good (0.61–0.80) and excellent (≥ 0.81). Count data were compared using Chi-squared test. The comparisons of Ktrans, Ve, Kep and ADC value of rectal cancer were performed by one-way analysis of variance (ANOVA) or rank-sum test (Kruskal–Wallis). The correlation between ADC value and quantitative DCE-MRI parameters (Ktrans, Ve and Kep) was analyzed by Spearman rank correlation test. The prediction performance of the model was evaluated by receiver operating characteristic (ROC) curve, and the prediction probability was quantified by the area under the curve (AUC), sensitivity, specificity and Youden index. Multivariate logistic regression analyses was conducted with DCE-MRI parameters and ADC values were used as covariables. *P* < 0.05 was considered to be statistically significant.

## Results

### Clinical-pathologic characteristics in mrEMVI-positive and -negative groups

**Fig. 2.**
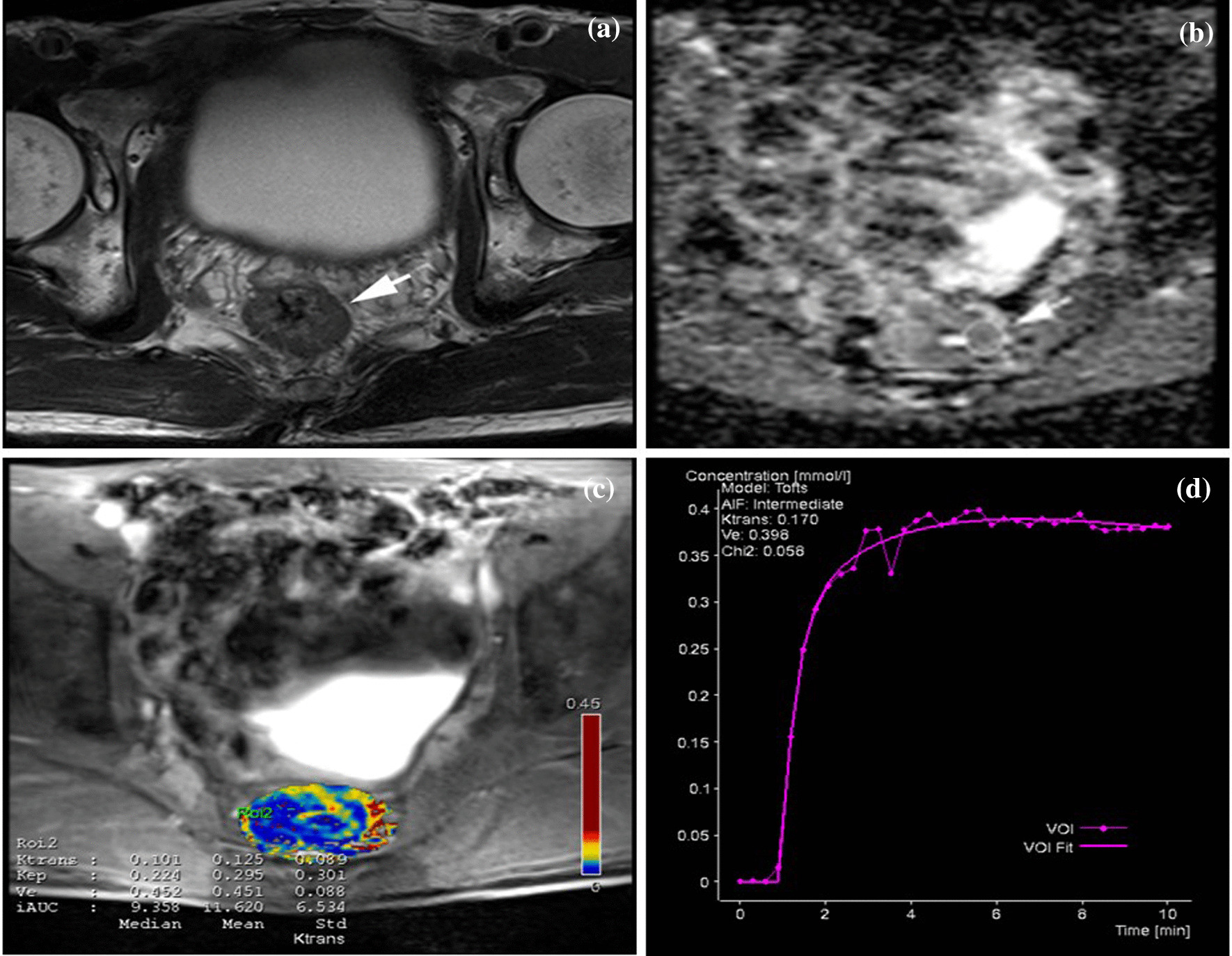
72-year-old man with Mr-detected extramural venous invasion (mrEMVI) negative rectal cancer. **a** High resolution T2WI (HR-T2WI) showed rectal wall thickening and no vessels adjacent to areas of tumor penetration. ADC (**b**) and perfusion (**c**) image of the rectal cancer segment showing ROI placement and DCE-MRI quantitative parameters values. **d** Time-signal intensity curve was platform curve

**Fig. 3.**
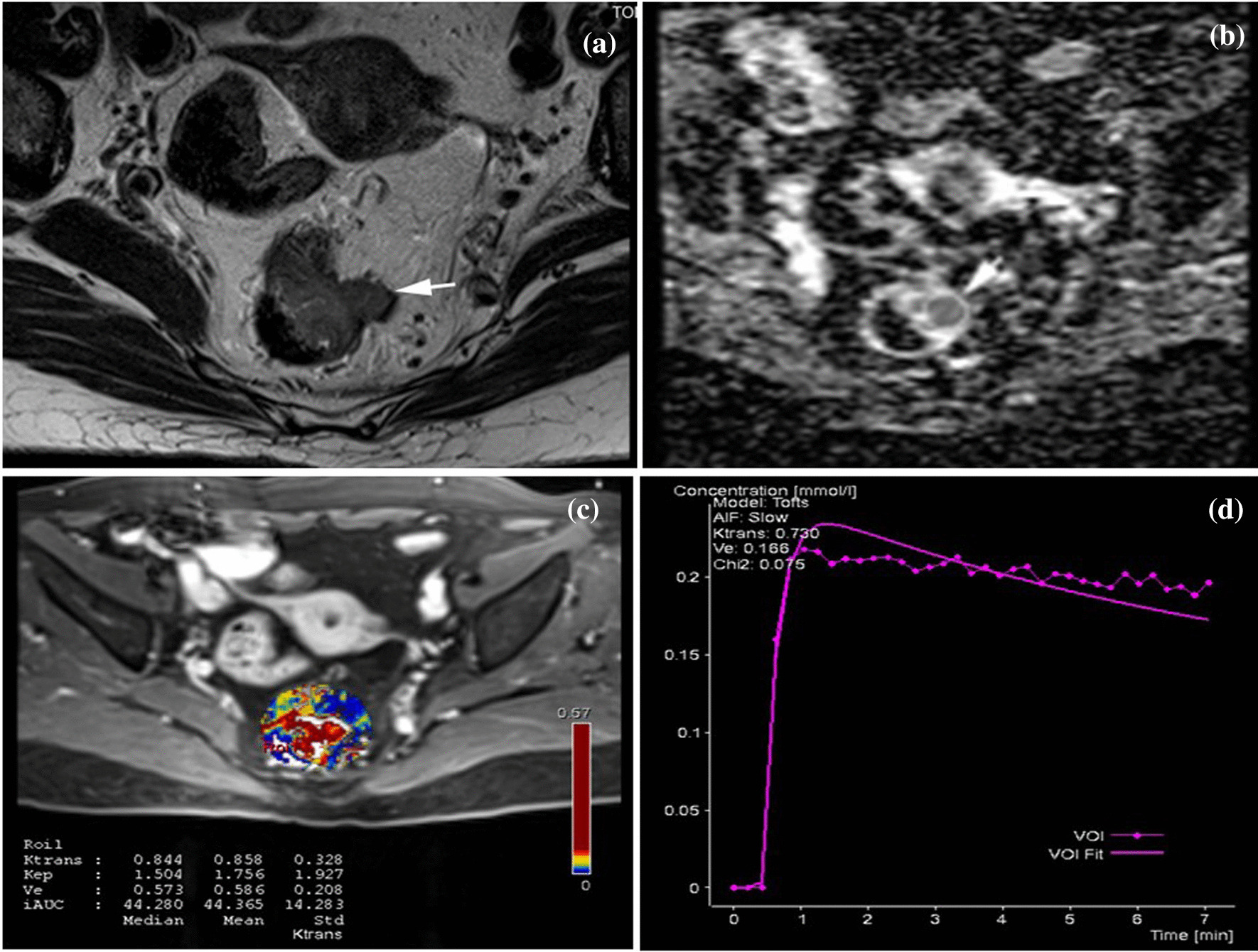
67-year-old woman with mrEMVI positive rectal cancer. **a** HR-T2WI showed rectal wall thickening and irregular nodular signal beyond the wall of rectum. ADC (**b**) and perfusion (**c**) image of the rectal cancer segment showing ROI placement and DCE-MRI quantitative parameters values. **d** Time-signal intensity curve was washout type

The pathological results of all 82 patients were rectal adenocarcinoma, with 58 were mrEMVI-negative (Fig. 2) and 24 were mrEMVI-positive (Fig. 3). Surgical methods: transabdominal perineal combined with radical resection of rectal cancer (Miles operation, 17 cases), transabdominal anterior resection of rectal cancer (Dixon operation, 56 cases), transabdominal resection of rectal cancer, artificial anus and distal closure (Hartmann operation, 9 cases). Infiltration depth, CIR, tumor location, histological grade, T stage, N stage and the average Ki67 expression in mrEMVI-positive and -negative groups were showed in Table 3.

**Table 3:** Comparison of clinical and histopathological characteristics between mrEMVI-positive and -negative groups

Parameters	Unit	All patients (n = 82)	mrEMVI ( +) (n = 24)	mrEMVI (-) (n = 58)	*P* value
Sex	M	47	12	35	0.465
	F	35	12	23	
Age	y	62.68 ± 12.35	64.58 ± 13.03	61.9 ± 12.09	0.374
CEA	ug/l	3.95 (2.2,9.97)	8.53 (2.87,26.6)	3.54 (2.17,7.0)	0.02
Infiltration depth	mm	13.55 (9.9,17.0)	15.0 (14.0,20.5)	12.0 (8.0,16.0)	< 0.01
CIR	%	73.23 ± 26.03	82.50 ± 21.41	69.39 ± 26.96	0.037
Location	Upper	17	4	13	0.824
	Middle	37	11	26	
	Low	28	9	19	
Histological grade	Well	18	1	17	< 0.01
	Moderately	48	14	34	
	Poorly	16	9	7	
T stage	T1	10	0	10	< 0.01
	T2	22	0	22	
	T3	32	11	21	
	T4	18	13	5	
*N stage*	N0	51	10	41	0.022
	N1	21	8	13	
	N2	10	6	4	
Ki67 expression	%	62.7 ± 22.5	71.0 ± 14.3	59.3 ± 24.5	0.031

Count data (sex, location, Histological grade, T stage, *N stage*) were analyzed by using Chi-squared test. CEA and Infiltration depth were showed as medians (IQR 25–75) and analyzed by Mann–Whitney test. Age, CIR and Ki67 expression was analyzed by ANOVA test

*CIR* Circum-involvement ratio

No significant differences were found for sex and age between mrEMVI-positive and -negative groups (*P* > 0.05). The two groups had statistically significant differences in serum CEA level, infiltration depth, CIR, gross classification, differentiation, T stage, N stage and Ki67 expression (*P* < 0.05). To be specific, in the mrEMVI-positive group, infiltration depth, CIR and Ki67 expression were much higher than those in the mrEMVI-negative group. The tumors in the mrEMVI-positive group were more poorly differentiated and had a higher proportion of lymph node metastasis than that in the mrEMVI-negative group (*P* < 0.05) (Table 3).

### Comparison between mrEMVI and pEMVI

Kappa test was used to assess consistency between MRI and pathology analysis of EMVI in rectal cancer. MRI examination and pathological examination had good consistency in evaluation of EMVI in patients with rectal cancer (Kappa = 0.775, *P* < 0.001) (Table 4).

**Table 4:** Comparison of MRI examination and pathological examination in the evaluation of EMVI status

MRI	Histopathology		
	pEMVI ( +)	pEMVI (-)	Total
mrEMVI (+)	22	2	24
mrEMVI (−)	6	52	58
Total	28	54	82

Kappa test were used to assess consistency results compared between MRI and pathology (Kappa = 0.775, *P* < 0.01)

### Differences in quantitative DCE-MRI parameters and ADC values between mrEMVI-positive and -negative groups

Interobserver agreements for quantitative parameters of DCE-MRI were assessed by ICC with 2-way random method between the two radiologists. Interobserver reproducibility was excellent for Ktrans, Ve, Kep and ADC values (ICC = 0.82, 95% CI: 0.73–0.88; ICC = 0.92, 95% CI: 0.88–0.95; ICC = 0.91, 95% CI: 0.87–0.94; and ICC = 0.82, 95% CI: 0.73–0.88, respectively).

**Fig. 4:**
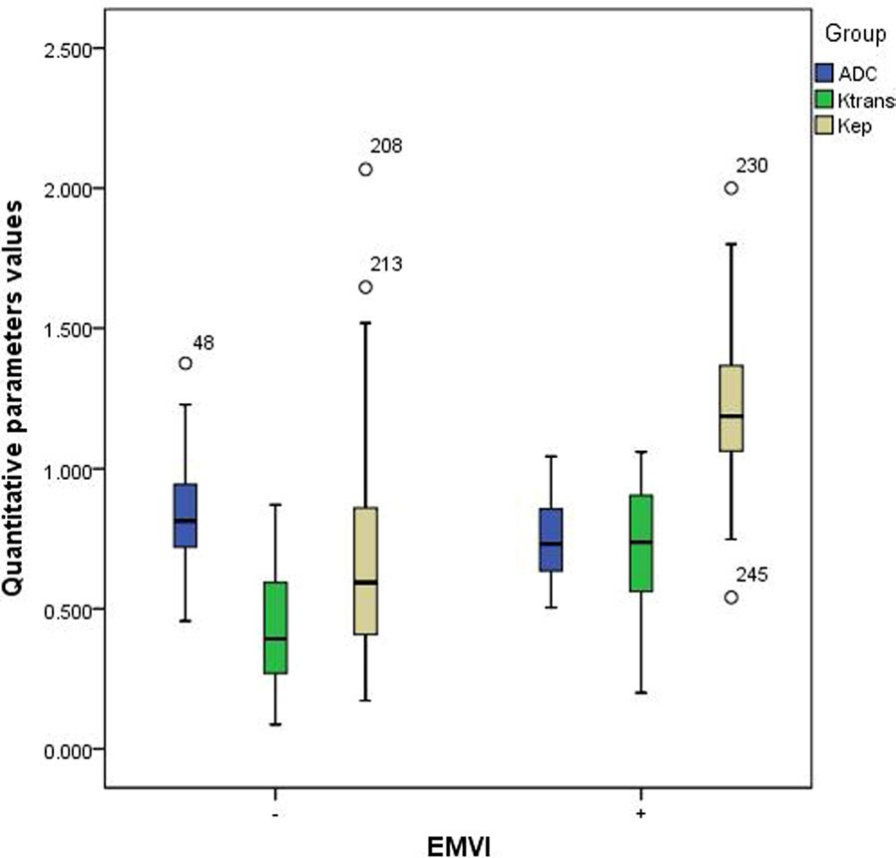
Box plot of the relationship of four quantitative parameters in mrEMVI –positive and –negative groups of rectal cancer

The comparisons of quantitative DCE-MRI parameters and ADC values between the mrEMVI-positive and -negative groups are summarized in Table 5. The results showed that, in the mrEMVI-positive group, the Ktrans and Kep values were significantly higher than those in the -negative group (*P* < 0.01), and the ADC values were significantly lower (*P* < 0.01) (Fig. 4), whereas Ve showed no significant difference between the two groups (*P* > 0.05).

**Table 5:** Comparison of quantitative DCE-MRI parameters and ADC values between mrEMVI-positive and -negative groups

mrEMVI	Ktrans	Ve	Kep	ADC
Positive	0.74 (0.56,0.91)	0.61 (0.51,0.7)	1.18 (1.06,1.37)	0.76 (0.66,0.86)
Negative	0.39 (0.27,0.59)	0.58 (0.49,0.75)	0.58 (0.41,1.06)	0.81 (0.72,0.95)
*P* value	< 0.01	0.878	< 0.01	0.04

The comparison of the four quantitative parameters (showed as medians: IQR 25–75) between mrEMVI-positive and -negative groups were performed by Mann–Whitney test

### Relationship of quantitative DCE-MRI parameters and ADC values

**Fig. 5:**
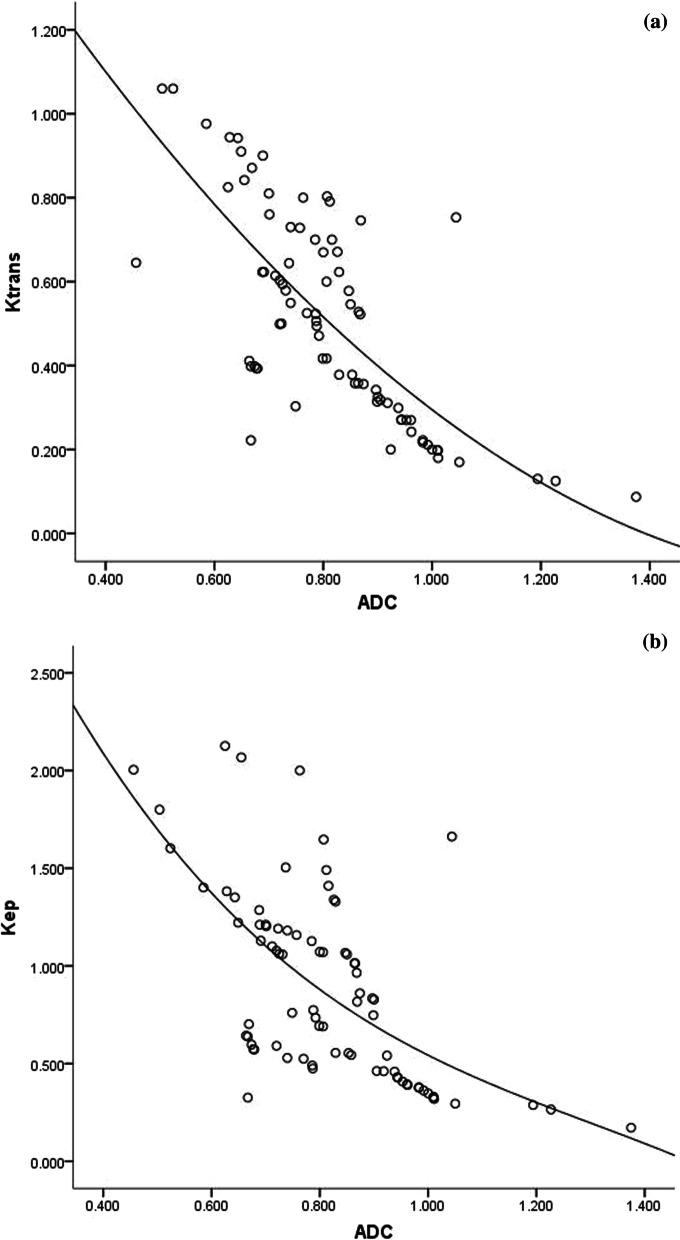
Correlation between ADC value and Ktrans (**a**), ADC value and Kep (**b**) for rectal cancer

A negative correlation was observed between the Ktrans vs ADC value (r = -0.724, *P* < 0.01) and Kep vs ADC value (r = -0.636, *P* < 0.01) in patients with rectal cancer (Fig. 5a,b). There is no obvious correlation between Ve vs ADC value (r = -0.073, *P* = 0.523) (Table 6).

**Table 6:** Relationship between quantitative DCE-MRI parameters and ADC values

Parameters	Ktrans	Ve	Kep
ADC			
r	− 0.724	− 0.073	− 0.636
*P* value	< 0.01	0.523	< 0.01

The correlation between ADC value and quantitative DCE-MRI parameters was analyzed by Spearman rank correlation test

### Prediction efficiency of pEMVI using Krans, Kep, and ADC values and Combined model

**Fig. 6:**
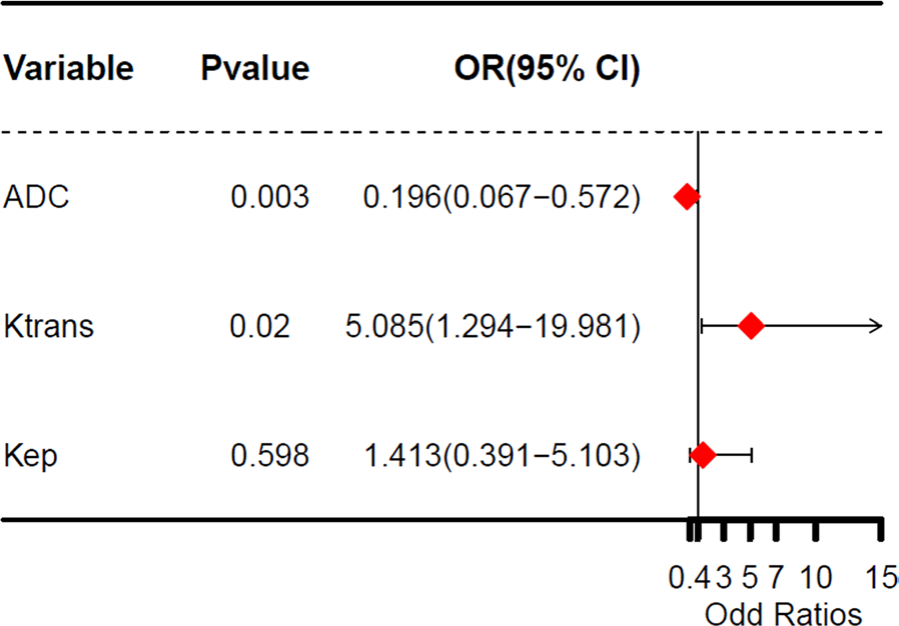
COX regression forest figure of pathologic EMVI (pEMVI) by using DCE-MRI quantitative parameters

**Fig. 7:**
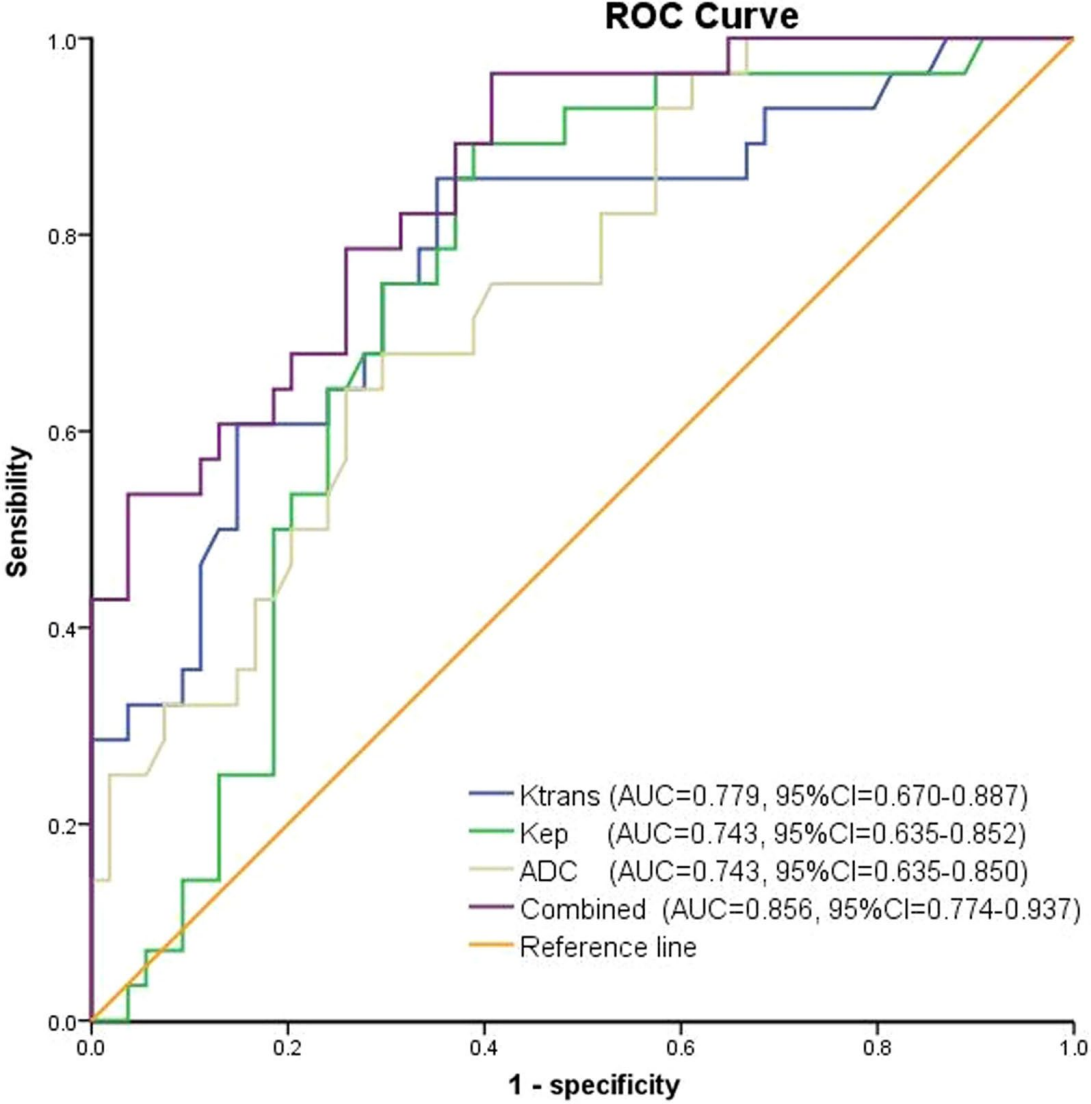
ROC curve of model established by DCE-MRI quantitative parameters and ADC values for the prediction of pEMVI in rectal cancer

Dualistic logistic regression analyses were conducted with Ktrans, Kep, and ADC values as covariables, and pEMVI as the dependent variable. The results showed that Ktran and ADC values were independently associated with the pEMVI with an odds ratio (OR) value of 5.085 and 0.196, respectively (Fig. 6). The combined prediction model based on quantitative DCE parameters and ADC values obtains the highest Youden index (0.557) among the quantitative parameters (Table 7). ROC analysis was used to evaluate the prediction efficiency of pEMVI. The results as follows: AUC was consistently higher in the combined model (0.856), followed by Ktrans (0.779), ADC (0.743) and Kep (0.743). The combined prediction model has a good prediction efficiency for pEMVI in rectal cancer, with an AUC value of 0.856 (95% CI: 0.774–0.937). (Fig. 7).

**Table 7:** Dualistic logistic regression analysis of Ktrans, Kep, ADC, and combined quantitative parameters, and their predictive impact on pathologic EMVI

Parameters	AUC	Sensibility	Specificity	Youden index	Odds ratio	*P* value
Ktrans	0.779	0.857	0.684	0.505	5.085	0.02
Kep	0.743	0.893	0.611	0.504	1.413	0.598
ADC	0.743	0.643	0.741	0.384	0.196	< 0.01
Combined	0.856	0.964	0.593	0.557	–	–

Combined: Ktrans + Kep + ADC. The prediction performance of the model was evaluated by ROC curve

## Discussion

Our study demonstrated that quantitative DCE-MRI and DWI parameters contribute to preoperative assessment of EMVI in rectal cancer. In the mrEMVI-positive group, serum CEA, infiltration depth, tumor CIR and Ki67 expression were higher than those in the mrEMVI-negative group. Compared with the EMVI-negative group, mrEMVI-positive patients were in higher T and N stages, and more poorly differentiated. In the relevant researches [11, 22], tumour size, extent invasion, differentiation and Ki67 expression were closely related to the malignancy and prognosis of rectal cancer. Other studies showed that mrEMVI was considered as a noninvasive and sensitive diagnostic biomarker for guiding treatment and predicting prognosis [6, 7]. Either before preoperative staging or neoadjuvant treatment, it is essential to identify the presence of mrEMVI in rectal cancer. Some literatures [3, 7, 8] have demonstrated that mrEMVI played as an independent predictor of lymph node metastasis, local recurrence, synchronous/metachronous distant metastases and disease-free/overall survival. To better clarify, we made a comparison between MRI and pathological examination for evaluation of EMVI in patients with rectal cancer, and found that they were in good agreement. A study revealed that mrEMVI detection before surgery might represent a surrogate of pathological EMVI [4]. Furthermore, in line with the results of studies based on pEMVI, mrEMVI made it possible to predict pEMVI preoperatively [6]. They provided evidence in support of fMRI as a reliable evaluation method for predicting EMVI before surgery.

We also compared quantitative DCE-MRI parameters and ADC values between the mrEMVI-positive and -negative groups. The results indicated that in the mrEMVI-positive group, the Ktrans and Kep values were significantly higher than those in the -negative group, while the ADC values were significantly lower in the -positive group. Generally, the higher the degree of malignancy of rectal cancer, the worse the prognosis, and the stronger capillary permeability was accompanied with higher Ktrans values [6, 14]***.*** This indicates that the Ktrans value may be positively correlated with the vascular invasion of rectal cancer. Similarly, higher Kep value indicates greater blood return to vasculature. Kep is only affected by the contrast concentration and fractional volumes in the tumour extravascular extracellular space and might thus more accurately reflect the tumour capillary [26]. It was reported that Ktrans and Kep permitted noninvasive estimation of rectal cancer angiogenesis [4]. Zhu et al. [6] summarized 79 patients to detected EMVI of locally advanced gastric cancer by DCE-MRI, which showed that EMVI-positive group had higher Ktrans and Kep than the negative group, while no correlation between Ve and mrEMVI was detected. They concluded that Ktrans, Kep, and ADC were independent predictors of EMVI in locally advanced gastric cancer. In contrast, some studies discriminated from our results. Chen et al. [11] analyzed 72 patients with rectal cancer to detect mrEMVI by DCE-MRI, which showed that mrEMVI-positive group got higher Ve values compared with the negative group. The possible reason might be that the complex microcirculation structure and heterogeneity in rectal cancer that caused uneven distribution of the blood flow leads to the variability of the Ve value accordingly [26].

In our study, ADC values in the mrEMVI positive group were significantly lower than that in the -negative group. Many studies [12, 27] have shown that as a sensitive image biomarker of rectal cancer, ADC value has a strong correlation with prognosis and staging of rectal cancer. As far as the grade of primary tumor was considered, lower ADC values were reported to be detected in more aggressive tumors [15]. A retrospective research [22] reported 77 cases of rectal cancer and found that the more advanced the T grade, the lower the ADC values were. These results were consistent with our study. Therefore, ADC values can be served as a promising, noninvasive imaging biomarker for evaluation of the aggressiveness of rectal cancer [22]. This was concluded by the fact that mrEMVI-positive rectal cancer was found to have significantly lower ADC values than mrEMVI-negative tumors.

Many literatures [6, 14, 22] showed that Ktrans, Kep and ADC values were closely related to aggressiveness and prognosis of various tumors, so we analyzed the relationship between DCE-MRI quantitative parameters and ADC values. A negative correlation was observed between the Ktrans vs ADC value and Kep vs ADC value in patients with rectal cancer. As far as we know, this is the first article to analyze the correlation between the DCE-MRI quantitative parameters and ADC values based on mrEMVI in rectal cancer. Meanwhile, it is proved that Ktrans, Kep, and ADC values are of great significance for the detection of mrEMVI in rectal cancer. Among the four quantitative parameters of MRI, Ktrans and ADC value were independently associated with mrEMVI of rectal cancer. A study [4] summarized 63 patients with rectal cancer to explore the correlations between DCE-MRI quantitative parameters and synchronous distant metastasis. They found that Ktrans, Kep, and Ve value were significantly higher in the lesions with distant metastasis than in the lesions without distant metastasis, and the Ktrans showed the highest AUCs among the DCE-MRI quantitative parameters, which is consistent with our study. Dijkhoff et al. [28] analyzed 18 literatures that evaluated DCE-MRI for tumour aggressiveness, primary staging and restaging after CRT. They indicated that DCE-MRI in rectal cancer was promising mainly for prediction and assessment of response to CRT, where a high pre-CRT Ktrans and a decrease in Ktrans were predictive for response. Our results consisted with these investigations. Among all the DCE-MRI parameters and ADC values, Ktrans and ADC value had the best performance, which were independent predictor of EMVI in rectal cancer. By construction of DCE-MRI and DWI combined predicting model, the combined diagnosis performance became better.

Several limitations are worth noting. First, the process of this measurement is cumbersome, low applicability may appear in other centers with other MRIs due to highly subjective ROI placing. For these reasons, prediction of EMVI in rectal cancer based on DCE-MRI has not yet have clinical impact on decision making. Second, for small tumors, this measurement of quantitative DCE-MRI parameters and ADC value may be more challenging. Third, the sample size in our study was relatively small, which may have affected statistical power. Whether there is a correlation between Ve and mrEMVI, more cases are needed for validation.

## Conclusions

In summary, the quantitative DCE-MRI parameters, Krans, Kep and ADC values played important roles in predicting EMVI of rectal cancer. Meanwhile, Ktrans and ADC value were the independent predictors of EMVI in rectal cancer. Quantitative DCE-MRI and DWI parameters can be used as powerful tools to predict EMVI, which has a good clinical application potential in the non-invasive preoperative individualized prediction of rectal cancer.

## Data Availability

All data generated or analyzed during this study are included in this published article.

## Abbreviations

ADC: Apparent diffusion coefficient
AUC: Area under the curve
CRT: Neoadjuvant chemoradiotherapy
DCE-MRI: Dynamic contrast-enhanced magnetic resonance imaging
CE-T1: Contrast-enhanced T1WI
DWI: Diffusion-weighted imaging
EMVI: Extramural venous invasion
FOV: Field of view
fMRI: Functional MRI
HR-T2WI: High resolution T2WI
ICC: Intraclass correlation coefficients
Kep: Reverse reflux rate constant
Ktrans: Volume transfer constant
mrEMVI: MRI-predicted extramural venous invasion
MRI: Magnetic resonance imaging
pEMVI: Pathologic extramural venous invasion
ROC: Receiver operating characteristic
ROI: Region of interest
Ve: Extracellular extravascular volume fraction
VOI: Volume of interest
